# Impact of chronic kidney disease on the prevalence of cardiovascular disease in patients with type 2 diabetes in Spain: PERCEDIME2 study

**DOI:** 10.1186/1471-2369-15-150

**Published:** 2014-09-16

**Authors:** Antonio Rodriguez-Poncelas, Gabriel Coll-De Tuero, Oriol Turrò-Garriga, Joan Barrot-de la Puente, Josep Franch-Nadal, Xavier Mundet-Tuduri, on behalf RedGDPS Study Group

**Affiliations:** EAP Anglès, Girona, España; IdIBGi, Girona, España; University of Girona, Girona, España; Institut Català de la Salut, EAP Salt, Girona, España; Institut Català de la Salut, EAP Raval Sud, Barcelona, España; Unitat de Suport a la Recerca Barcelona Ciutat, Institut Universitari d’Investigació en Atenció Primària Jordi Gol (IDIAP Jordi Gol), Barcelona, Spain; Research Unit, Edifici Mancomunitat1, Parc Hospitalari Martí i Julià, Dr. Castany s/n 17190 Salt, Girona, Spain; Universitat Autònoma de Barcelona, Bellaterra, Spain

**Keywords:** Type 2 diabetes, Chronic kidney disease, Cardiovascular disease

## Abstract

**Background:**

The presence of chronic kidney disease (CKD) in type 2 diabetes mellitus (T2DM) increases the risk of cardiovascular disease (CVD) regardless of the presence of traditional cardiovascular risk factors. There is controversy about the impact of each of the manifestations of CKD on the prevalence of CVD, whether it is greater with decreased estimated glomerular filtration rate (eGFR) or increased urine albumin creatinine ratio (UACR).

**Methods:**

This study is a national cross-sectional study performed in primary care consults. We selected participants of both sexes who were aged 40 years or older, had been diagnosed with T2DM and had complete information on the study variables recorded in their medical records. The participants were classified according to eGFR : ≥ 60; 45–59; 30–44; <30 mL/min/1.73 m^2^ and UACR : < 30; 30–299; ≥300 mg/gr. The results were adjusted to compare the prevalence of CVD across all categories.

**Results:**

A total of 1141 participants were included. Compared to participants with eGFR > 60 mL/min/1.73 m^2^ those with eGFR between 30–44 mL/min/m^2^, (OR = 2.3; 95% CI, 1.4-3.9); and eGFR < 30 mL/min/1.73 m^2^ (OR = 4.1 95% CI 1.6-10.2) showed increased likelihood of having CVD. Participants with UACR ≥ 30 mg/g compared to participants with UACR < 30 mg/g increased significantly the likelihood of having CVD, especially with UACR above 300 mg/g, (OR = 1.6; 95% CI 1.1-2.4 for UACR = 30–299 mg/g; OR = 3.9; CI 1.6-9.5 for UACR ≥ 300 mg/g).

**Conclusion:**

The decrease in eGFR and increase in UACR are independent risk factors that increase the prevalence of CVD in participants with T2DM and these factors are independent of each other and of other known cardiovascular risk factors. In our study the impact of mild decreased eGFR in T2DM on CVD was lower than the impact of increased UACR. It is necessary to determine not only UACR but also eGFR for all patients with T2DM, both at the time of diagnosis and during follow-up, to identify those patients at high risk of cardiovascular complications.

## Background

Chronic kidney disease (CKD) is a serious public health problem that increases the risk of overall mortality, cardiovascular disease (CVD), and progression to end-stage renal disease regardless of the presence of traditional cardiovascular risk factors [1–3].

The presence of microalbuminuria in diabetes mellitus has been considered one of the first clinical signs of diabetic nephropathy. Microalbuminuria can progress to macroalbuminuria, subsequently leading to a decrease in the estimated glomerular filtration rate (eGFR), and finally to end-stage renal disease [4]. This process was originally described for type 1 diabetes and may be different in patients with type 2 diabetes mellitus (T2DM).

Controversy exists regarding the impact of each of the manifestations of CKD on the prevalence of CVD, whether it would be greater with increased urine albumin creatinine ratio (UACR) or a decreased eGFR. Several epidemiological studies show that increased UACR is associated with a higher prevalence of CVD; this association is observed even with low levels of UACR, and the progression of UACR influences the clinical outcome [5, 6], particularly regarding cardiovascular complications [7, 8]. It has been found that people with decreased eGFR and normal UACR have a lower CVD risk than individuals with increased UACR and normal eGFR [7, 9, 10]. In some studies in patients with T2DM, it has been observed that increased UACR and decreased eGFR are associated with the same prevalence of CVD, although the increase in UACR seems to relate more strongly to cardiovascular death [11, 12].

Most clinical practice guidelines advise determining not only UACR but also eGFR, as decreased eGFR with normal UACR is common in T2DM [13–15]. The decrease in eGFR and increase in UACR are independently associated with increased overall mortality [11] and high prevalence of CVD [16, 17], which are even higher if they are both present.

This study was designed to determine the relationship of the decrease in eGFR and the increase in UACR with the prevalence of CVD in participants with T2DM being treated in primary care settings in Spain.

## Methods

### Study design and population

A detailed description of the methodology used has been described previously [18]. This study was a national cross-sectional study performed in primary care consults. The participants included both genders and were over 40 years old. Moreover, the participants were diagnosed with TDM2, and they had all of the variables necessary for the study included in their clinical history. Each researcher included 15 participants who met the inclusion criteria, a maximum of 3 participants per day. The participants were selected by convenience sample of the first 3 patients with diabetes each day who came to the consult for any reason and met the inclusion criteria until complete the number of participants per researcher.

The information for each participant was collected in the period from February to July 2011. The Ethical and Clinical Investigation Committee of the Institut d’ Assistència Sanitària (IAS) in Salt, Girona, Spain approved the study. To obtain the necessary data for this study from the clinical history of participants, all participants provided written informed consent.

### Variables and procedure

We followed a study protocol to ensure correct collection of variables. The following variables were collected during the visit: age, gender, ethnicity, weight, height, body mass index (BMI) was calculated as weight in kilograms divided in meters squared; obesity was defined as BMI ≥ 30 kg/m^2^, abdominal waist circumference, cardiovascular risk factors. Cardiovascular diseases was collected through the medical record as a history of stroke (ischaemic cerebrovascular disease) included only symptomatic brain infarction, and did not include silent brain infarction, transient ischaemic attack or brain haemorrhage. Coronary heart disease included a previous history of myocardial infarction, angina pectoris, the presence of coronary interventions or the presence of ECG abnormalities suggestive of coronary heart disease. Peripheral arterial disease was diagnosed by an ankle-brachial pressure index of < 0.9 and/or two absent foot pulses. Diabetes mellitus was defined as fasting glucose ≥ 126 mg/dL or non-fasting glucose ≥ 200 mg/dL or use of glucose-lowering drugs. Hypertension was defined as systolic blood pressure of 140 mmHg or greater, diastolic blood pressure of 90 mmHg or greater, or use of antihypertensive medications irrespective of blood pressure. Hyperlipidemia was defined as total cholesterol ≥ 250 mg/dL, LDL cholesterol > 155 mg/dL, HDL cholesterol < 40 mg/dL in men and < 48 mg/dL in women, triglycerides > 150 mg/dL or pharmacologic lipid lowering treatment. Cigarette smoking was defined as:never/past/current.

The following clinical and analytical measurements were collected: DM2 duration, arterial pressure (average of the last three determinations), drugs taken by the patient at the time the data were collected (antidiabetic, antihypertensives, hypolipemiant, antiplatelet, and anticoagulants). Basal glycaemia, glycosylated haemoglobin (HbA1c), haemoglobin, total cholesterol, LDL cholesterol, HDL cholesterol, non-HDL cholesterol, triglycerides, serum creatinine after overnight fasting for at least 12 hr, and the UACR in a urine sample collected in the first morning urine specimen, using the most recent value of the last 12 months.

In this study, the presence of CKD was based on KDOQI criteria as follows: patients with eGFR < 60 mL/min/1.73 m^2^ or the presence of renal damage if UACR values were elevated (UACR ≥ 30 mg/g). CKD- EPI equation was used to calculate the eGFR [19]. Albuminuria was defined as a UACR of 30 mg/g or more. Microalbuminuria was defined as a UACR ranging from 30 to 299 mg/g, and macroalbuminuria was defined as a UACR of 300 mg/g or more. Two determinations of eGFR and UACR, in a period of three or more months, must be made. The cases that did not fulfil two determinations were excluded from the final analysis of the study.

The participants were classified according to simplified Kidney Disease Improvement Global Outcomes (KDIGO 2012) Guidelines [20], eGFR : ≥ 60; 45–59; 30–44; <30 mL/min/1.73 m^2^ and UACR : < 30; 30–299; ≥300 mg/gr.

To compare the prevalence of CVD across all categories, the results were adjusted by age and sex (Model 1); by age, sex, hypertension, dyslipidaemia, smoking, and BMI (Model 2); and by age, sex, hypertension, dyslipidaemia, smoking, BMI, HbA1c, and duration of diabetes (Model 3). Normal eGFR values were considered to be equal to or higher than 60 mL/min/1.73 m^2^; decreased eGFR values were less than 60 mL/min/1.73 m^2^; normal UACR values were below 30 mg/g; and increased UACR values were above 30 mg/g.

### Statistical analysis

Measurements of central tendency and dispersion were used to perform a descriptive analysis of the quantitative variables studied. Absolute and relative frequencies were used for qualitative variables. The patient groups were established according to the UACR and eGFR values. The differences in demographic and clinical characteristics between groups were calculated by comparison of means in quantitative variables, parametric or nonparametric according to the normal distribution of each variable, and contingency tables for qualitative variables, with a Yates correction when necessary. The prevalence of CVD was established for each of the eGFR and UACR categories. To determine the variables associated with CVD in each category, different logistic regression models were adjusted to control for potential confounders. We performed also a multivariate analysis considering the eGFR and UACR as continuous variables from point considered normal.

The results were expressed as absolute numbers, percentages, medians, standard deviations, odds ratios (ORs), and 95% CI. The statistical significance was set at 0.05 when contrasting hypotheses. The data analysis and processing were performed using the SPSS 17.0 statistical program for Windows.

## Results

During the recruitment, 1279 participants were included in the study, but 138 (10.8%) participants were excluded because of incomplete or erroneous data. To rule out bias attributable to participants excluded from the study, we compared the two groups and we found no significant differences. The final sample size was 1141 participants.

Table 1 shows clinical and metabolic characteristics of the 1141 participants. Participants with GFR < 60 mL/min/m^2^ compared to participants with GFR ≥ 60 mL/min/m^2^ were more frequently older, had longer diabetes duration and had a higher rate of hypertension. When comparing participants with UACR ≥ 30 mg/g and participants with UACR <30 mg/g, those had longer diabetes duration, higher systolic blood pressure, triglycerides, higher rate of hypertension and lower eGFR.

**Table 1:** **Clinical and metabolic characteristics of participants with type 2 diabetes (n = 1,141)**

Variables	ALL	eGFR ≥60	eGFR <60	UACR < 30	UACR ≥ 30
**n**	1141	761	380	987	154
**Age** (years); mean (SD)	66.8 (11.3)	63.8 (10.9)	72.7 (9.4)	66.4 (11.2)	68.7 (11.1)
**Gender** (men) ,%	60.6	80.3	50.7	60.7	59.7
**Duration diabetes** (years), mean (SD)	9.1 (6.7)	8.4 (6.5)	10.2 (6.9)	8.8 (6.5)	10.4 (7.4)
**Body mass index** (kg/m^2^) mean (SD)	30.3 (5.2)	30.6 (5.4)	29.5 (4.8)	30.1 (5.2)	31.1 (5.5)
**Abdominal waist circumference** (cm); mean (SD)	100.0 (17.1)	99.9 (16.9)	99.9 (18.0)	99.5 (17.3)	101.8 (16.9)
**Obesity** (%)	46.5	48.9	41.3	45.9	49.4
**HbA1c;** mean (SD)	7.3 (1.3)	7.6 (1.4)	7.2 (1.2)	7.2 (1.3)	7.7 (1.4)
**Smoking** (%)	13.8	39.4	50.5	42.5	47.4
**Hyperlipidemia** (%)	67,9	67.1	68.9	67.5	69.5
**Hypertension** (%)	75.1	70.0	83.2	72.9	83.8
**Systolic Blood Pressure** (mmHg); mean (SD)	134.5 (13.2)	134.4 (13.0)	134.8 (13.5)	134.1 (12.7)	137.4 (15.5)
**Diastolic Blood Pressure** (mmHg); mean (SD)	77.0 (9.1)	78.1 (8.7)	74.7 (9.4)	77.1 (9.0)	76.7 (9.7)
**Cholesterol total** (mmol/L); mean (SD)	4.70 (0.93)	187.1 (37.3)	176.8 (36.1)	183.8 (36.8)	183.3 (40.3)
**Cholesterol HDL** (mmol/L); mean (SD)	1.29 (0.35)	49.1 (13.1)	45.8 (13.8)	48.3 (13.4)	46.0 (12.5)
**Cholesterol LDL** (mmol/L); mean (SD)	2.79 (0.83)	110.4 (31.9)	103.1 (31.9)	108.4 (31.8)	105.0 (33.6)
**Cholesterol non HDL** (mmol/L); mean (SD)	3.49 (0.91)	137.9 (36.2)	131.2 (35.1)	135.4 (35.4)	137.5 (39.4)
**Tryglicerides** (mmol/L); mean (SD)	1.69 (0.96)	149.8 (91.2)	154.4 (94.2)	146.5 (83.7)	182.3 (130.7)
**Plasmatic creatinina** (mg/dl); mean (SD)	0.93 (0.3)	0.78 (0.13)	1.2 (0.3)	0.9 (0.3)	1.0 (0.4)
**Glomerular filtration ratio mL/min/1,73 m** ^**2**^; mean (SD)	79.9 (23.5)	89.6 (19.7)	47.6 (9.6)	76.8 (25.7)	68.3 (27.7)
**Urine albumin-creatinine ratio** (mg/g); mean (SD)	39.2 (144.3)	17.2 (35.2)	28.3 (52.7)	7.5 (6.2)	107 (66.2)

eGFR: estimated glomerular filtration rate according to CKD-EPI in mL/min/1.73 m^2^; UACR: urinary albumin creatinin rate in mg/gr.

Table 2 shows the number and percentage of participants classified by GFR and UACR according to adapted KDIGO Guidelines [20].

**Table 2:** **Distribution of chronic kidney disease markers by KDIGO criteria**

eGFR	UACR <10 mg/gr	UACR 10-29 mg/gr	UACR ≥ 30 mg/gr	Total
**>90**	246 (34.0)	90 (34.1)	40 (26.0)	376
**60-90**	255 (35.3)	85 (32.2)	45 (29.2)	385
**45-60**	46 (6.4)	20 (7.6)	26 (16.9)	92
**30-45**	164 (22.7)	62 (23.5)	36 (23.4)	262
**<30**	12 (1.7)	7 (2.7)	7 (4.5)	23
**TOTAL**	723	264	154	1141

KDIGO: Kidney Disease Improvement Global Outcome.

Table 3 shows the characteristics of participants according to the different combinations of UACR and eGFR. Participants with decreased eGFR were older; they had a higher prevalence of hypertension, and a higher percentage of them were receiving antihypertensive treatment. These participants had less peripheral arterial disease and higher prevalences of coronary heart disease and stroke. In the group of participants with increased UACR, we observed higher levels of HbA1c and a greater number of participants being treated with insulin. No interactions between eGFR and albumiburia were observed.

**Table 3:** **Patient characteristics according to the glomerular filtration rate and urinary albumin creatinine ratio**

Variables	Values	eGFR (nm)/	eGFR (nm)/	eGFR ↓/	eGFR ↓/	P value UACR↑ vs	P value eGFR ↓ vs
		UACR(nm)	UACR ↑	UACR (nm)	UACR ↑	UACR(nm)	eGFR (nm)
**N**	1141	674	85	311	69	154 vs 987	380 vs 761
**Age** (years) (SD)	66.8 (11.3)	63.7 (11.0)	64.3 (10.2)	72.4 (9.3)	74.1 (9.7)	0.048	<0.001
**Gender** (men,%)	691 (60.6)	345 (51.0)	44 (48.2)	254 (81.7)	51 (73.9)	0.823	<0.001
**T2DM Duration** (years) (SD)	9.1 (6.7)	8.4 (6.4)	8.7 (6.3)	9.6 (6.5)	12.6 (8.1)	0.007	<0.001
**BMI** (kg/m^2^) (SD)	30.3 (5.2)	30.5 (5.4)	31.2 (5.4)	29.3 (4.5)	30.9 (5.6)	0.058	0.004
**BMI ≥30** kg/m^2^ (%)	529 (46.4)	327 (48.4)	45 (52.9)	126 (40.5)	31 (44.9)	0.424	0.016
**Waist circumference** (cm) (SD)	100.0 (17.1)	99.8 (16.8)	100.4 (17.5)	99.0 (18.3)	103.6 (16.1)	0.118	0.514
**HbA1c** mean (SD)	7.3 (1.3)	7.3 (1.3)	7.8 (1.5)	7.1 (1.2)	7.5 (1.1)	<0.001	0.052
**Smoking** n (%)						0.467	<0.001
*Current*	158 (13.8)	96 (14.2)	18 (21.2)	37 (11.9)	7 (10.1)		
*Past-smoker*	334 (29.3)	164 (24.3)	22 (25.9)	122 (39.2)	26 (37.7)		
*Never*	649 (56.9)	416 (61.5)	45 (52.9)	152 (48.9)	36 (52.2)		
**Insulin** n (%)	281 (24.6)	159 (23.5)	26 (30.6)	65 (20.9)	31 (44.9)	<0.001	0.725
**Oral antidiabetic agents** n (%)	965 (84.6)	586 (86.7)	77 (90.6)	249 (80.1)	53 (76.8)	0.953	0.001
**Oral antidiabetic agents + insulin** n (%)	210 (18.4)	124 (18.3)	23 (27.1)	43 (13.8)	20 (29.0)	0.001	0.261
**Hyperlipidemia** n (%)	773 (67.7)	228 (33.7)	22 (25.9)	93 (29.9)	25 (36.2)	0.621	0.540
**Hypertension** n (%)	849 (74.4)	465 (68.8)	68 (80.0)	255 (82.0)	61 (88.4)	0.004	<0.001
**Antihypertensive treatment** n (%)	866 (75.9)	466 (68.9)	74 (87.1)	263 (84.6)	63 (91.3)	<0.001	<0.001
**Systolic Blood Pressure** (mmHg) (SD)	134.5 (13.2)	134.0 (35.4)	137.8 (14.9)	134.4 (12.8)	136.9 (16.4)	0.018	0.539
**Diastolic Blood Pressure** (mmHg) (SD)	77.0 (9.1)	78.1 (8.8)	78.2 (9.3)	74.7 (9.2)	74.8 (9.8)	0.892	<0.001
**Cholesterol total** (mmol/L) (SD)	183.7 (37.2)	186.9 (36.8)	189.1 (41.4)	177.0 (35.8)	176.2 (37.9)	0.853	<0.001
**Cholesterol HDL** (mmol/L) (SD)	135.7 (35.9)	137.4 (35.4)	142.8 (41.4)	131.2 (35.0)	131.0 (35.9)	0.514	0.004
**Cholesterol LDL** (mmol/L) (SD)	107.9 (32.1)	110.5 (35.4)	109.2 (33.5)	103.8 (31.6)	99.6 (33.2)	0.211	<0.001
**Cholesterol non HDL** (mmol/L) (SD)	48.0 (13.5)	49.5 (13.3)	46.2 (11.5)	45.8 (13.8)	45.7 (13.7)	0.055	<0.001
**Tryglicerides** (mmol/L) (SD)	151.3 (92.2)	146.4 (88.8)	177.3 (105.4)	146.8 (71.6)	188.4 (157.0)	<0.001	0.296
**Lipid-lowering therapy** n, (%)	738 (64.7)	413 (61.1)	60 (70.6)	215 (69.1)	50 (72.5)	0.060	0.012
**Plasmatic creatinina** (mg/dl) (SD)	0.92 (0.3)	0.78 (0.13)	0.78 (0.14)	1.17 (0.23)	1.31 (0.45)	<0.001	<0.001
**Glomerular filtration ratio, mL/min/1,73 m2** (SD)	75.6 (26.1)	89.8 (19.7)	88.3 (19.9)	48.5 (9.1)	43.6 (10.6)	<0.001	<0.001
**Urine albumin-creatinine ratio** (mg/g) (SD)	20,9 (42,2)	7.2 (5.9)	96.7 (61.3)	8.0 (6.7)	119.8 (70.1)	<0.001	0.002
**Coronary heart disease** (%)	163 (14.3)	67 (9.9)	9 (10.6)	68 (21.9)	19 (27.5)	0,137	<0.001
**Peripheral arterial disease** (%)	91 (8.0)	32 (4.7)	8 (9.4)	33 (10.6)	18 (26.1)	<0.001	<0.001
**Stroke** (%)	72 (6.3)	31 (4.6)	5 (5.9)	29 (9.3)	7 (10.1)	0.416	0.002

p-value bettween interactions low eGFR and albuminuria,> 0.05 in all cases.

nm: normal; eGFR: estimated glomerular filtration rate according to CKD-EPI in mL/min/1.73 m^2^; UACR: urinary albumin creatinin rate in mg/gr; BMI: body mass index.

**Figure 1:**
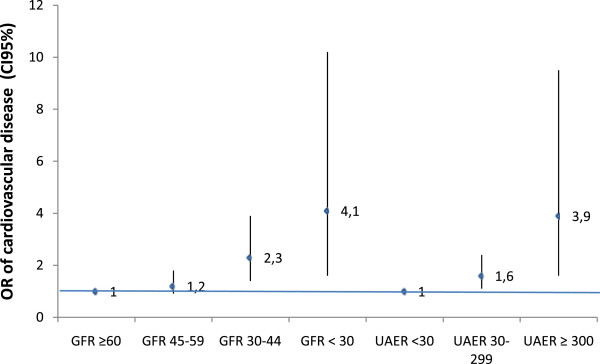
**Odds ratio of cardiovascular disease in Type 2 Diabetes Mellitus according to eGFR and UACR.**

Table 4 and Figure 1 shows the results for the prevalence of CVD based on the unadjusted model and after adjusting the results according to Models 1, 2 and 3. Compared to participants with GFR ≥ 60 mL/min/m^2^ those with GFR < 60 mL/min/m^2^ (Model 3) increased significantly the likelihood of having CVD, especially with eGFR less than 45 mL/min/m^2^, (OR = 1,2; 95% CI 0.9-1.8 for eGFR 45–59 mL/min/m^2^; OR = 2.3; CI 1.4-3.9 for eGFR 30–44 mL/min/m^2^ and OR = 4.1; CI 1.6-10.2 for eGFR < 30 mL/min/m^2^). Compared to participants with UACR < 30 mg/g those with UACR ≥ 30 mg/g (Model 3) increased significantly the likelihood of having CVD, especially with UACR above 300 mg/g, (OR = 1.6; 95% CI 1.1-2.4 for UACR = 30–299 mg/g; OR = 3.9; CI 1.6-9.5 for UACR ≥ 300 mg/g). Both, decreased eGFR and increased UACR, had higher likelihood of having CVD.

**Table 4:** **Multivariate analysis of chronic kidney disease impact on the prevalence of cardiovascular disease**

	Unadjusted model (OR; CI95%)	Model 1 (OR; CI95%)	Model 2 (OR; CI95%)	Model 3 (OR; CI95%)
Glomerular filtration rate (eGFR)				
eGFR ≥ 60 mL/min/1.73 m^2^	1	1	1	1
eGFR 45–59 mL/min/1.73 m^2^	2.07 (1.5-2.8)	1.2 (0.9-1.7)	1.2 (0.9-1.8)	1.2 (0.9-1.8)
eGFR 30–44 mL/min/1.73 m^2^	4.6 (2.9-7.3)	2.5 (1.5-4.1)	2.3 (1.4-3.8)	2.3 (1.4-3.9)
eGFR < 30 mL/min/1.73 m^2^	8.4 (3.6-19.9)	4.3 (1.7-10.7)	4.1 (1.6-10.2)	4.1 (1.6-10.2)
For each unit below 60 mL/min/1.73 m^2^	1.072 (1.06-1.09)	1.045 (1.03-1.06)	1.042 (1.02-1.06)	1.043 (1.02-1.06)
UACR				
Normoalbuminuria	1	1	1	1
Microlbuminuria (30–299 mg/gr)	1.9 (1.4-2.7)	1.8 (1.2-2.6)	1.8 (1.3-2.7)	1.6 (1.1-2.4)
Macroalbuminuria (≥300 mg/gr)	5.0 (2.3-11.1)	4.6 (1.9-10.9)	4.2 (1.8-10.2)	3.9 (1.6 -9.5)
For each unit above 29 mg/gr	1.001 (0.99-1.01)	1.001 (0.99-1.01)	1.001 (0.99-1.01)	1.001 (0.99-1.01)
UACR (normal) + GFR (normal)	1	1	1	1
UACR ↑ and eGFR ↓	3.3 (1.5-7.3)	2.3 (1.0-5.3)	2.4 (1.0-5.4)	2.2 (0.9-5.2)

GFR: estimated glomerular filtration rate. eGFR (normal): ≥ 60 mL/min/1,73 m2. eGFR↓: < 60 mL/min/1,73 m2.

UACR: Urine albumin/creatinine ratio . UACR (normal) < 30 mg/g. UACR ↑: ≥ 30 mg/g.

Normoalbuminuria: UACR < 30 mg/g. Microalbuminuria: UACR = 30–299 mg/g . Macroalbuminuria: UACR >300 mg/g.

Model 1 Adjusted for age and sex.

Model 2 Adjusted for age, sex, hypertension, dislipidaemia, tobacco and Body Mass Index.

Model 3 Adjusted for age, sex, hypertension, dislipidaemia , tobacco, Body Mass Index , HbA1c and T2DM duration.

## Discussion

In this study, we observed that in participants with T2DM treated in primary care settings, decreased eGFR and increased UACR were associated with a higher rate of cardiovascular disease. The results of our study show a higher prevalence of CVD (30.9% vs. 8.5%) than in the study by Yokoyama et al. [17] The older age of the participants (67 vs. 58 years), higher BMI (30 vs. 24 kg/m^2^), differences in systolic blood pressure (135 vs. 128 mmHg) and diastolic blood pressure (77 vs. 75 mmHg), and increased waist circumference could explain these differences.

The increase in UACR relates to the presence of renal disease and CVD [21] and indicates the probable existence of generalised endothelial damage [22]. An association has been established between increased UACR levels and increased coronary risk [23] and stroke [24]. In Brantsma et al. [7] study, the increase in UACR was a better predictor for the development of CVD than the decrease in eGFR; subjects with moderately decreased eGFR and normal UACR had no increased risk of CVD, whereas participants with moderately decreased eGFR and increased UACR had an increased risk of CVD. Notably, the number of subjects with eGFR < 45 mL/min/1.73 m^2^ was very small, and the significance of eGFR on CVD risk could have been underestimated. When comparing the risk of cardiovascular and all cause mortality by assessing different degrees of eGFR and UACR, for participants with increased UACR and eGFR greater than 60 mL/min/1.73 m^2^,the initial stages of CKD, it was found that the risk of CVD was higher in participants with macroalbuminuria than in participants with microalbuminuria. Decreased eGFR in these stages was not associated with an increased CVD risk. However, CVD risk increases progressively with decreasing eGFR below 60 mL/min/1.73 m^2^.

Association between a moderate decreased eGFR and increased CVD risk has been observed in some epidemiological studies [1, 25, 26]; however, this relationship is not present, or is very weak, in other studies [27, 28]. Go et al. [29] showed a gradual association between reduced eGFR and increased risk of CVD death and hospitalisation, regardless of other known risk factors, history of CVD, and the presence of proteinuria. This risk was evident when eGFR was less than 60 mL/min/1.73 m^2^ and increased significantly for subjects whose eGFR was less than 45 mL/min/1.73 m^2^ [11].

The clinical factors associated with decreased eGFR and increased UACR may be common, but could also be different. The decrease in eGFR and increase in UACR are risk factors for developing CVD; it is unknown whether this association is independent of the presence of other known cardiovascular risk factors. It has been suggested that the increase in UACR and decrease in eGFR might be renal manifestations of endothelial dysfunction and systemic atherosclerosis, respectively. It is likely that increased UACR and decreased eGFR could be markers of different pathological processes [30].

Cardiovascular and renal complications share common risk factors, such as hypertension, hyperlipidaemia, obesity, hyperglycaemia, left ventricular hypertrophy, increased inflammatory markers, increased vascular permeability, and disorders of the coagulation system and fibrinolysis [1, 29–32]. The existence of CKD may indicate that these risk factors have been present for a long period of time, causing a greater or lesser prevalence of CVD related to their duration.

According to other studies [12, 33], we observed a low prevalence of CVD in our participants with mild decreased eGFR although the relationship between eGFR as a continuous variable and the prevalence of CVD is strong. In contrast to the study by Solini et al. [34], we did not observe a significant increase in the prevalence of CVD in the group with decreased eGFR and increased UACR, most likely because of the small number of participants who had this combination in our study, which determined that the results of this group are most likely not relevant. Another possibility is that in this group of participants , most have an eGFR and UACR in closer to normal areas, with lower cardiovascular risk.

Our study showed different results from those of the meta-analysis by Lee et al. [24]; the group of participants with decreased eGFR had a higher prevalence of stroke than the group with increased UACR. Both markers of renal dysfunction, decreased eGFR and increased UACR, are associated with a higher prevalence of CVD in diabetic patients; the presence of one or both markers of renal dysfunction would indicate the presence of a vascular disorder, and the presence of CVD itself causes a deterioration of renal function [35, 36]. In this study, the OR of developing CVD increased significantly below eGFR 45 mL/min/1.73 m^2^ and particularly with levels below 30 mL/min/1.73 m^2^.

Among the study’s limitations, it should be mentioned that it is a cross-sectional study; therefore, a causal relationship cannot be established. Although the study variables were collected from the medical records of participants by the researcher, the possibility of clinical underdiagnosis cannot be ruled out. Only a small number of participants had UACR ≥ 300 mg/ and/or eGFR < 30 mL/min/1.73 m^2^, which limits the ability of the study to assess the impact of eGFR and UACR in the most advanced stages of CKD. The generalisation of our findings may be limited; all the participants in this study had T2DM and were willing to participate voluntarily in the study. However, we believe that this population is representative of the type of care that is performed in primary care settings. Finally, the serum creatinine level and UACR were not measured centrally, which may affect the accuracy of the results. Among the strengths of the study, it should be noted that this is a national study with a large sample of patients with T2DM, in which family physicians were heavily involved in the management of the disease; and at least 2 of 3 measurements had to be altered over a period of at least 3 months to establish the diagnosis of chronic kidney disease.

## Conclusion

The decrease in eGFR and increase in UACR are independent risk factors that increase the prevalence of CVD in patients with T2DM. In our study the impact of mild decreased GFR in T2DM on CVD was lower than the impact of increased UACR. These results suggest that it is necessary to determine both had higher likelihood of having CVD and UACR for all patients with T2DM—at the time of diagnosis and during follow-up—to identify patients at high risk of cardiovascular complications, to establish the proper preventive measures, and to intensify control in patients with T2DM and CKD, in an effort to reduce cardiovascular morbidity and mortality.

## Abbreviations

CKD: Chronic kidney disease
CVD: Cardiovascular disease
eGFR: Estimated glomerular filtration rate
T2DM: Type 2 diabetes mellitus
UACR: Urinary albumin creatinine ratio
BMI: Body mass index.
